# Chemotherapy combined with immunotherapy vs. chemotherapy: comparison of safety and efficacy in adjuvant therapy for intrahepatic cholangiocarcinoma

**DOI:** 10.3389/fonc.2026.1764663

**Published:** 2026-06-22

**Authors:** Lu Yang, Haijing Zheng, Zhaolong Pan, Dongyang Li, Yunlong Cui, Huikai Li, Qiang Wu, Tianqiang Song, Qiang Li, Wei Zhang

**Affiliations:** Department of Hepatobiliary Surgery, Liver Cancer Prevention and Treatment Research Center, Tianjin Medical University Cancer Institute & Hospital, National Clinical Research Center for Cancer, Tianjin’s Clinical Research Center for Cancer; Tianjin Key Laboratory of Digestive Cancer, Key Laboratory of Cancer Prevention and Therapy, Tianjin, China

**Keywords:** adjuvant chemotherapy, adverse reactions, immunotherapy, intrahepatic cholangiocarcinoma, survival

## Abstract

**Background:**

The aim of this retrospective study was to investigate the efficacy and safety of different regimes among patients with intrahepatic cholangiocarcinoma (ICC).

**Methods:**

The data of 232 patients (2011-2023) at the Tianjin Medical University Cancer Institute&Hospital were analyzed. Patients were divided into 5 different groups: non-AT group (underwent surgical treatment alone), gemcitabine plus oxaliplatin (GEMOX), gemcitabine plus immunotherapy, capecitabine monotherapy and capecitabine plus immunotherapy.

**Results:**

Significant differences were observed in both RFS (p = 0.045) and OS (p = 0.0084) between CH group (GEMOX and capecitabine) and non-AT group. No significant difference was observed in RFS (p = 0.73) or OS (p = 0.47) between capecitabine and GEMOX. However, GEMOX group had higher incidence of grade 3-4 adverse reactions(n=7, 17.1%). When comparing the CH and gemcitabine plus immunotherapy and capecitabine plus immunotherapy group, no significant differences were observed in RFS or OS. In subgroup analysis, patients with CA19-9>39 U/ml and ALP>100 U/L may benefited from chemotherapy combined with immunotherapy.

**Conclusion:**

Compared to GEMOX, capecitabine showed no significant difference in prognosis but exhibited a more favorable safety profile. Although the addition of immunotherapy did not confer further benefit in the overall cohort, it was associated with improved efficacy in patients with CA19-9 >39 U/ml or ALP >100 U/L. These findings require confirmation in larger studies.

## Introduction

Intrahepatic cholangiocarcinoma (ICC) is the second most common primary liver malignancy following hepatocellular carcinoma (1). Studies have shown that ICC accounts for 10% to 15% of primary liver cancers (2). ICC has gained an increasing attention over the past 10-20 years, primarily due to its morbidity and mortality worldwide (3). Surgical resection is the only radical treatment. However, because of its highly aggressive and metastatic nature, ICC has a very high recurrence rate after surgery, ranging from 40% to 80% (4), and a low 5-year overall survival (OS) rate after resection, ranging from 25% to 40% (5). Given high recurrence rates and poor OS, there is a need to improve the postoperative treatment for ICC. Postoperative therapy can be seen as the measure to prevent and delay the recurrence of disease.

Based on the BILCAP (6) (phase III clinical trial) and the American Society of Clinical Oncology (ASCO) (7) guidelines, patients with resected biliary tract cancer should be recommended adjuvant capecitabine for 6 months. The National Comprehensive Cancer Network (NCCN) guidelines also support the use of adjuvant chemotherapy for biliary tract cancer (7). However, it is critical to note that BILCAP enrolled a mixed BTC population that included not only ICC but also gallbladder cancer and extrahepatic cholangiocarcinoma, each with distinct biological behaviors. Data specific to ICC remain limited, and while dedicated phase III trials of adjuvant therapy in resected ICC are currently ongoing—including a trial directly comparing capecitabine versus GEMOX—but their results are not yet available; therefore, the optimal adjuvant regimen remains undefined. Consequently, equipoise persists between the guideline-recommended capecitabine and the GEMOX (gemcitabine plus oxaliplatin) (NCT02548195) regimen, which remains widely adopted in Asian practice based on institutional experience and retrospective data in biliary malignancies (8). The role of immunotherapy in this setting is even less defined.

This study therefore aimed to investigate the necessity of postoperative adjuvant chemotherapy for ICC, compare the efficacy and safety of capecitabine versus GEMOX, and explore the role of immunotherapy in a real-world cohort.

## Data and methods

### Data sources

The data of 232 patients diagnosed with ICC, who underwent radical resection between January 2011 and October 2023 at the Tianjin Medical University Cancer Institute and Hospital, were retrospectively analyzed. In the study, the median follow-up duration was 21 months.

**Figure 1:**
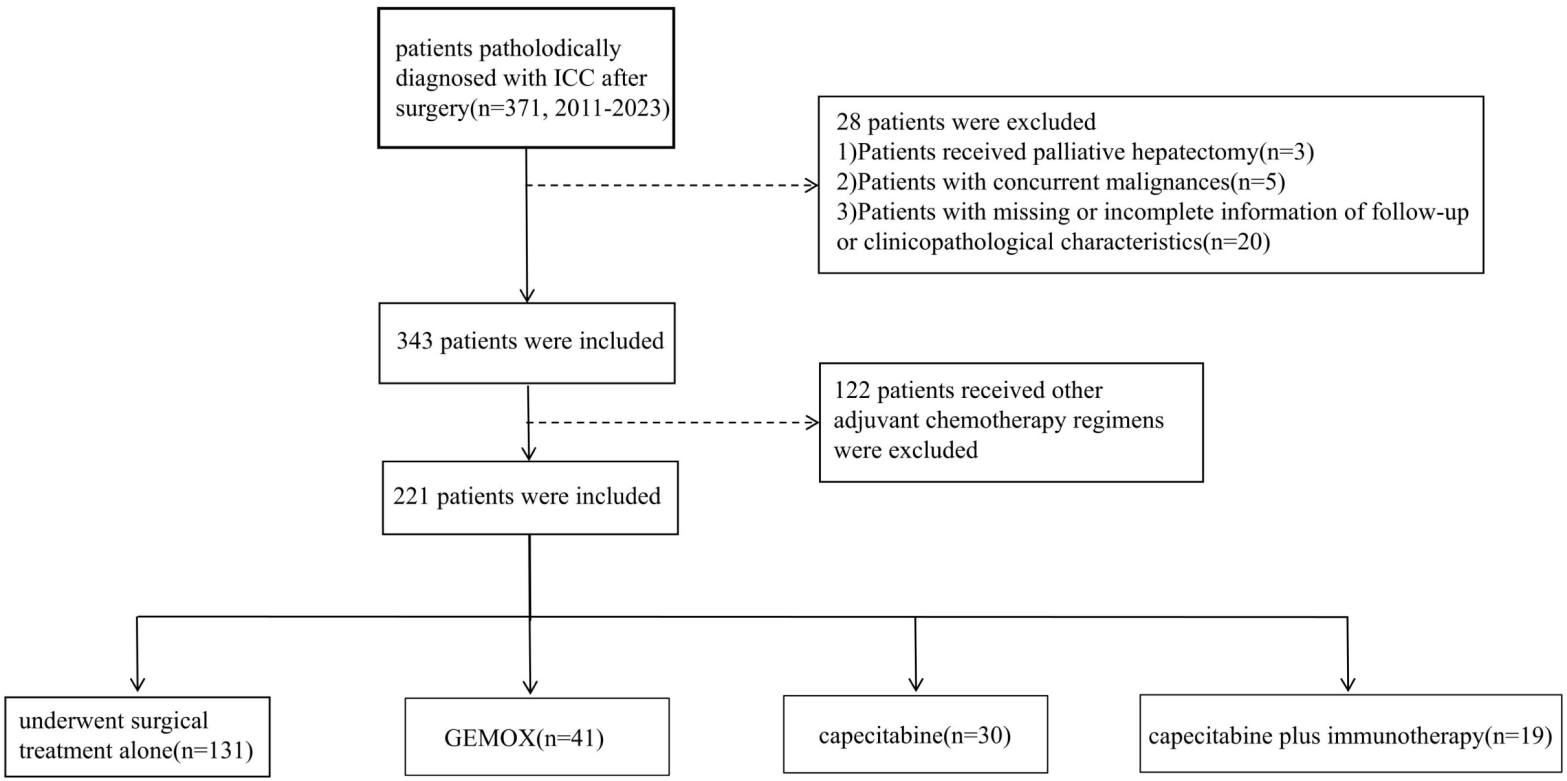
Flow chart of the patient enrolling process. GEMOX, gemcitabine plus oxaliplatin.

The inclusion criteria were as follows: (1) age≥18 years old; (2) underwent radical resection of intrahepatic cholangiocarcinoma; (3) pathological confirmation of ICC. The exclusion criteria were as follows: (1) nonradical resection (pathologically confirmed positive resection margin; (2) perioperative death; (3) concurrent malignancies; (4) missing or incomplete information. A flow diagram for data selection is shown in **Figure 1**.

All tumors of ICC were staged according to the TNM classification system of the International Union Against Cancer (8th edition) and the AJCC (8th edition). Liver function was assessed using the Child-Pugh classification. This study was conducted in accordance with the Declaration of Helsinki (2013 revision). Ethical approval was granted by the Ethics Committee of Tianjin Medical University Cancer Institute & Hospital, and the requirement for informed consent was waived.

### Surgical treatment and adjuvant chemotherapy

The surgical method of hepatectomy was selected according to the tumor site, relationship of the tumor with the liver and surrounding important blood vessels, liver cirrhosis, and residual liver volume. Patients included in this study received the following four adjuvant therapy regimens: gemcitabine plus oxaliplatin (GEMOX), gemcitabine plus immunotherapy, capecitabine monotherapy and capecitabine plus immunotherapy. Immune checkpoint inhibitors include toripalimab, sintilimab, tislelizumab, pembrolizumab, druvalumab. Among them, gemcitabine plus immunotherapy and capecitabine plus immunotherapy were referred as the chemotherapy combined with immunotherapy group (CHI group). GEMOX and capecitabine were referred to as the chemotherapy group (CH group). The specific dose of chemotherapeutic drugs was individually determined by a clinical oncologist and was adjusted according to the situation of the patients.

### Follow-up

Survival data, including Overall Survival (OS) and Recurrence-free Survival (RFS), were collected until December 30, 2024. Data were censored at the last follow-up for surviving patients. Overall survival was defined as the time from surgery to death from any cause, with censoring on the last date the patient was documented to be alive. RFS was defined as the time interval from surgery to first recurrence. RFS was considered as the primary endpoint. Secondary endpoints included OS, safety profiles. Patients were followed up every three months for the first two years and every 4-6 months thereafter, and tests included serum carcinoembryonic antigen (CEA), carbohydrate antigen19-9 (CA19-9), liver ultrasonography, computed tomography (CT) or magnetic resonance imaging (MRI). The incidence and severity of adverse events and serious adverse events, as well as the frequency and distribution of abnormal laboratory values, were measured in accordance with the Common Terminology Criteria for Adverse Events (CTCAE, version 5.0).

### Statistical analysis

The SPSS Statistics (version 29.0.1.0) and R (version 4.4.2) software were used for analysis. Student’s t-test was used for continuous variables with normal distribution, Mann-Whitney U-test was used for continuous variables with non-normal distribution, and basic clinical indicators were analyzed by Chi-square test or Fisher’s exact test for categorical variables. Kaplan-Meier survival curve and log-rank test were used to analyze RFS and OS. A 1:1 or 1:2 match between groups was performed using the nearest-neighbor matching method to be within a range of 0.2 SD. A P-value of <0.05 was considered statistically significant. The cutoff for CA19-9 was defined as >39 U/ml, the upper limit of normal. For alkaline phosphatase (ALP), the optimal cutoff for predicting recurrence was determined using X-tile software (version 3.6.1; Yale University), identifying >100 U/L as the best discriminant value.

## Results

### 1 Comparison of clinicopathological factors between CH group and non-AT group.

The demographic and clinical characteristics of patients are shown in **Table 1**. A total of 131 patients were included in the non-AT group who received only surgical treatment. A total of 41 patients were in the GEMOX group. There were 30 patients in the capecitabine group. We compare the clinicopathological factors between ICC patients treated with two chemotherapy regimens (GEMOX, capecitabine) and non-AT group. There were statistical differences in variables such as gender (p = 0.010), age (p = 0.016) and tumor size (p = 0.039). The propensity score was derived from covariates that were significant at p < 0.05 in univariable analysis. Following matching, the groups were comparable on all baseline variables (**Table 1**, all p > 0.05) (**Supplementary Figures 1**, **2**).

**Table 1:** The clinicopathological characteristics of ICC patients treated with two chemotherapy regimens (GEMOX, capecitabine) and non-AT.

Variable	Non-AT before PSM (n=131)	Non-AT after 1:1 PSM (n=71)	CH group (n=71)	P1	Before PSM SMD	P2	After PSM SMD
Gender (Male vs Female)	80/51(61.1%)	31/40(43.7%)	30/41(42.3%)	**0.010**	0.381	0.865	0.134
Age (≥55 vs <55 years)	111/20(84.7%)	53/18(74.6%)	50/21(70.4%)	**0.016**	0.314	0.573	0.039
Tumor size (cm) (median, range)	4.5(3-7)	5(4-7)	5(4.3-7.5)	**0.039**	0.220	0.483	0.071
Number (solitary vs multiple)	112/19(85.5%)	62/9(87.3%)	62/9(87.3%)	0.720	0.055	1.000	0.196
TNM (III/IV vs I/II)	40/91(30.5%)	27/44(38.0%)	28/43(39.4%)	0.201	0.182	0.863	0.000
Lymph node metastasis (positive vs negative)	22/109(16.8%)	17/54(23.9%)	13/58(18.3%)	0.786	0.088	0.411	0.067
CA19-9 (U/mL) (median, range)	45.615(13.11-258.3)	56.68(13.66-379.4)	36.22(15.59-247.13)	0.988	0.017	0.606	-0.002
HBeAg (positive vs negative)	38/93(29.0%)	18/53(25.4%)	21/50(29.6%)	0.932	0.012	0.573	0.036
Ascites (yes vs no)	7/124(5.3%)	5/66(7.0%)	1/70(1.4%)	0.322	0.334	0.211	0.000
Child Pugh (B vs. A)	6/125(4.6%)	5/66(7.0%)	3/68(4.2%)	1.000	0.096	0.716	0.076
γ-GT (U/L) (median, range)	53(29-117)	64(29-128)	44(30-115)	0.765	0.082	0.419	0.150
ALP (U/L) (median, range)	93(70-129)	105(77-150)	88(70-125)	0.684	-0.040	0.152	0.159
ALT (≥39 vs <39 U/ml)	33/98(25.2%)	20/51(28.2%)	11/60(15.5%)	0.111	0.268	0.068	0.093
AST (≥40 vs <40 U/ml)	22/109(16.8%)	15/56(21.1%)	8/63(11.3%)	0.292	0.175	0.111	0.222
Albumin (g/L) (median, range)	42.9(39.7-46.1)	42.9(38.9-46.5)	43.9(40.5-46.1)	0.515	0.032	0.511	-0.054

Bold text hinted that these variables were statistically significant.

P^1^: comparison between CH group and non-AT group before PSM.

P^2^: comparison between CH group and non-AT group after 1:1 PSM.

ICC, intrahepatic cholangiocarcinoma; GEMOX, gemcitabine plus oxaliplatin; non-AT, patients who received only surgical treatment; CH group, chemotherapy group; CA19-9, carbohydrate antigen 19-9; HBeAg, hepatitis B e antigen; γ-GT, γ-glutamyl transferase; ALP, alkaline phosphatase; ALT, alanine aminotransferase; AST, aspartate aminotransferase; PSM, propensity score matching. SMD, standardized mean difference.

### 2 Survival analysis of the CH group and non-AT group.

**Figure 2:**
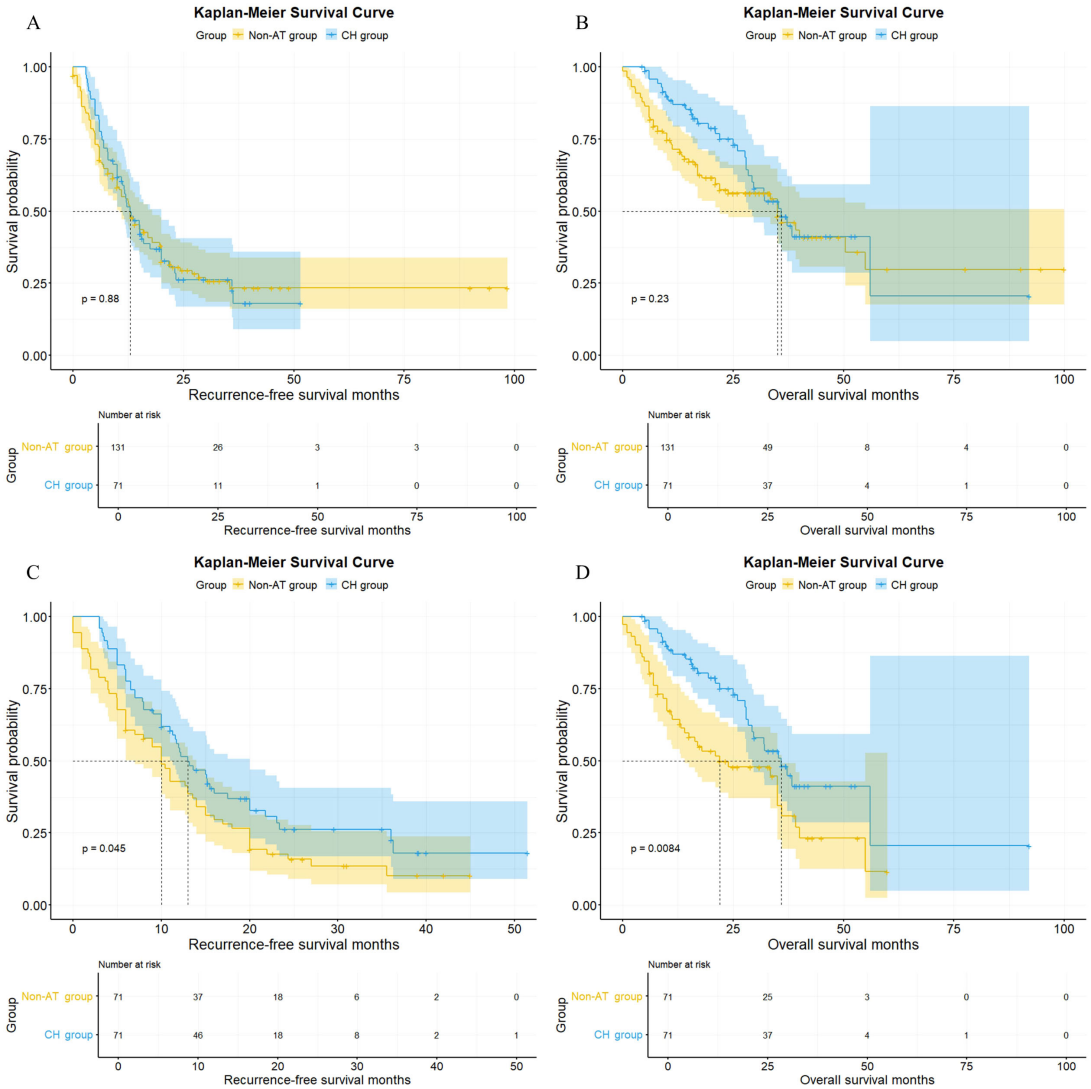
Kaplan-Meier analyses for overall survival (OS) and recurrence-free survival (RFS) between the CH group (n=71) and non-AT group (n=131), the CH group (n=71) and non-AT group (n=71) after PSM. **(A)** RFS in the non-AT group versus the CH group before PSM; **(B)** OS in the non-AT group versus the CH group before PSM; **(C)** RFS in the non-AT group versus the CH group after 1:1 PSM; **(D)** OS in the non-AT group versus the CH group after 1:1 PSM. CH group, chemotherapy group; non-AT group, patients who received only surgical treatment; PSM, propensity matching scores.

The study revealed no statistically significant differences in RFS and OS between the CH group and non-AT group before PSM. Conversely, following PSM, significant differences were observed in both RFS (CH group vs non-AT group median RFS: 13.0 vs 10.0 months, p = 0.045) and OS (CH group vs non-AT group median OS: 35.9 vs 22.0 months, p = 0.0084) between the two groups (**Figure 2**).

### 3 Comparison of clinicopathological factors and survival analysis of ICC patients treated with GEMOX and capecitabine.

**Figure 3:**
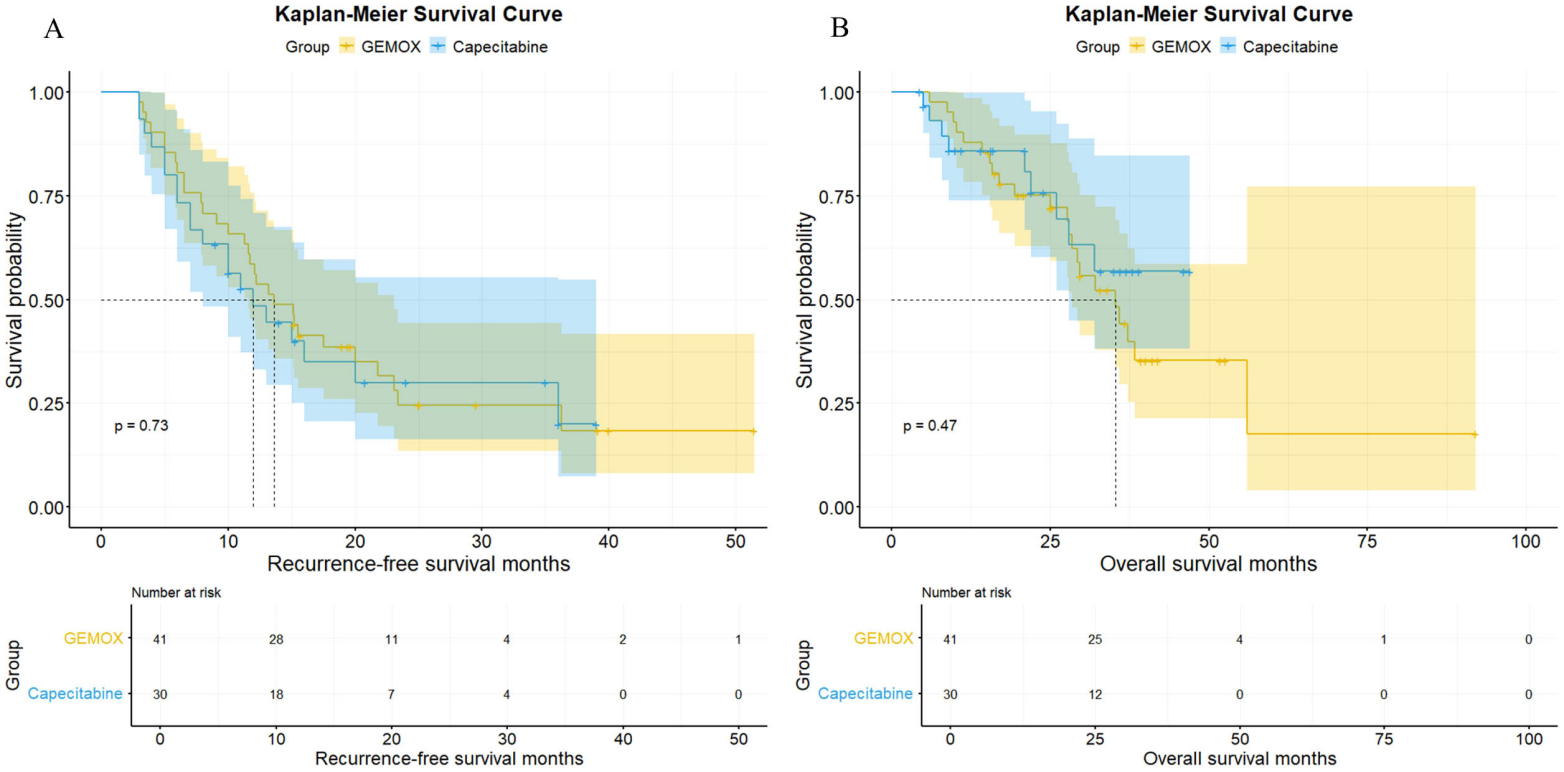
Kaplan-Meier analyses for recurrence-free survival (RFS) and overall survival (OS) based on different chemotherapy regimens (GEMOX group, n=41; capecitabine group, n=30). **(A)** RFS in the capecitabine group versus the GEMOX group; **(B)** OS in the capecitabine group versus the GEMOX group. GEMOX, gemcitabine plus oxaliplatin.

The demographic and clinical characteristics of patients are shown in **Table 2**. A total of 41 patients who received GEMOX; of which, 17 were men and 24 were women, with a median tumor size of 5cm. A total of 30 patients received capecitabine, of which 13 were men and 17 were women, with a median tumor size of 6 cm. No significant difference was observed in baseline data between the two groups (all p > 0.05). No significant difference was observed in RFS (GEMOX vs capecitabine median RFS: 13.63 vs 12.0 months, p = 0.73) or OS (capecitabine median OS has not been reached, GEMOX median OS: 35.27 months, p = 0.47) between the two groups (**Figure 3**).

**Table 2:** The clinicopathological characteristics of ICC patients treated with GEMOX and capecitabine.

Variable	Capecitabine (n=30)	GEMOX (n=41)	P
Gender (Male vs Female)	13/17(43.3%)	17/24(41.5%)	0.875
Age (≥55 vs <55 years)	25/5(83.3%)	28/13(68.3%)	0.076
Tumor size (cm) (median, range)	6.00(4.45-7.00)	5(4-9)	0.889
Number (solitary vs multiple)	26/4(86.7)	36/5(87.8%)	1.000
TNM (III/IV vs I/II)	10/20(33.3%)	18/23(43.9%)	0.368
Lymph node metastasis (positive vs negative)	3/27(10%)	10/31(24.4%)	0.121
CA19-9 (U/ml) (median, range)	34.54(15.515-562.4)	39.18(15.815-322.15)	0.929
HBeAg (positive vs negative)	8/22(26.7%)	13/28(31.7%)	0.646
Ascites (yes vs no)	0/30(0%)	1/40(2.4%)	1.000
Child Pugh (B vs. A)	0/30(0%)	1/40(2.4%)	0.359
γ-GT (U/L) (median, range)	45.5(27.75-105)	44(30.5-128.5)	0.633
ALP (U/L) (median, range)	85.5(69.75-121.75)	90(69.5-129)	0.714
ALT (≥39 vs <39 U/ml)	3/27(10%)	8/33(19.5%)	0.446
AST (≥40 vs <40 U/ml)	2/28(6.7%)	6/35(14.6%)	0.504
Albumin (g/L) (median, range)	43.6(40.5-46.15)	44.1(40.35-46.15)	0.907

ICC, intrahepatic cholangiocarcinoma; GEMOX, gemcitabine plus oxaliplatin; CA19-9, carbohydrate antigen 19-9; HBeAg, hepatitis B e antigen; γ-GT, γ-glutamyl transferase; ALP, alkaline phosphatase; ALT, alanine aminotransferase; AST, aspartate aminotransferase.

### 4 Safety of the GEMOX and capecitabine groups.

**Figure 4:**
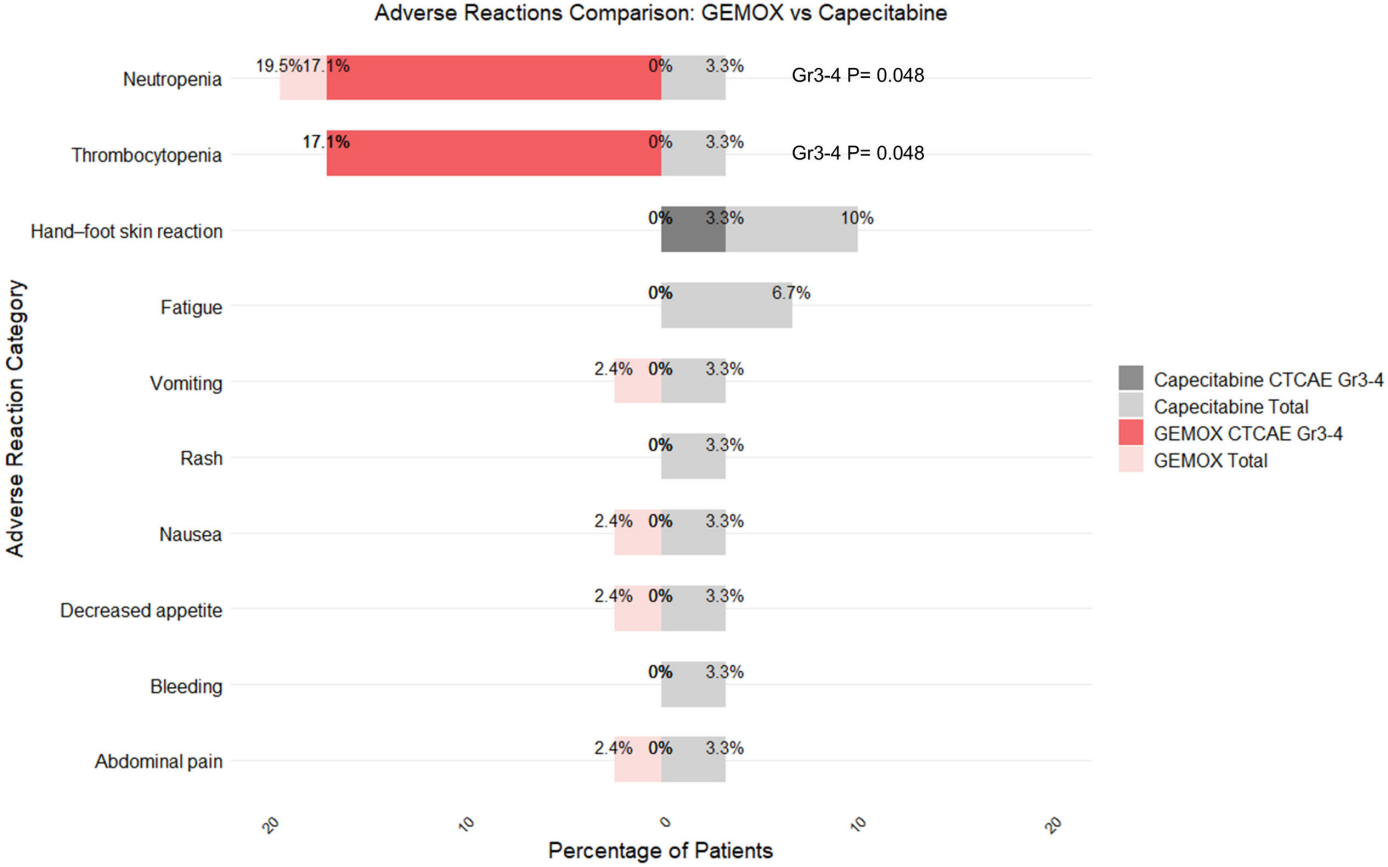
Pyramid plot of adverse events. GEMOX, gemcitabine plus oxaliplatin.

A total of 71 patients with ICC received GEMOX and capecitabine for safety analysis, and all adverse events were evaluated (**Figure 4**; **Supplementary Table 1**).

In the GEMOX group, neutropenia was the most common adverse event (n=8; 19.5%), followed by thrombocytopenia (n=7; 17.1%). Moreover, seven patients developed severe thrombocytopenia and neutropenia with Grade 3-4. In the capecitabine group, hand-foot skin reaction was the most common adverse event (n=3; 10%), and fatigue was the second highest incidence of adverse reactions (n=2; 6.7%). One of the patients developed severe hand and foot reactions with grade 3-4. Overall, GEMOX was more likely to cause severe myelosuppression, while the capecitabine regimen showed higher safety.

### 5 Comparison of clinicopathological factors between CH group and CHI group.

The demographic and clinical characteristics of patients are shown in **Table 3** (**Supplementary Figures 3**, **4**). A total of 71 patients were included in the CH group. A total of 30 patients were included in the CHI group. We compare the clinicopathological factors between the two group. Notably, we included chemotherapy regimen as a variable to ensure comparability between the two groups. There was statistical difference in variable such as gender (p = 0.011). After propensity score matching using variables with p < 0.05 in univariable analysis, no significant differences were observed in baseline characteristics (**Table 3**, all p > 0.05).

**Table 3:** Comparison of clinicopathological factors between CH group and CHI group.

Variable	CH group before PSM (n=71)	CH group after 1:2 PSM (n=60)	CHI group (n=30)	P1	Before PSM SMD	P2	After PSM SMD
Gender (Male vs Female)	30/41(42.3%)	30/30(50.0%)	21/9(70.0%)	**0.011**	0.605	0.071	0.240
Age (≥55 vs <55 years)	50/21(70.4%)	44/16(73.3%)	24/6(80.0%)	0.320	0.239	0.488	0.108
Tumor size (cm) (median, range)	5.0(4.3-7.5)	5(4.075-7)	5(4-6.75)	0.379	-0.231	0.586	-0.041
Number (solitary vs multiple)	62/9(87.3%)	52/8(86.7%)	23/7(76.7%)	0.297	0.252	0.230	0.239
TNM (III/IV vs I/II)	28/43(39.4%)	30/30(50.0%)	15/15(50.0%)	0.327	0.211	0.453	0.037
Lymph node metastasis (positive vs negative)	13/58(18.3%)	12/48(20.0%)	9/21(30.0%)	0.193	0.255	0.290	0.065
CA19-9 (U/ml) (median, range)	36.22(15.59-247.13)	37.89(15.94-930.1)	80.69(17.78-571.15)	0.519	-0.196	0.737	-0.297
HBeAg (positive vs negative)	21/50(29.6%)	19/41(31.7%)	9/21(30.0%)	0.966	0.009	0.872	0.189
Ascites (yes vs no)	1/70(1.4%)	1/59(1.7%)	3/27(10.0%)	0.143	0.286	0.206	0.161
Child Pugh (B vs. A)	3/68(4.2%)	3/57(5.0%)	1/29(3.3%)	1.000	0.050	1.000	0.161
γ-GT (U/L) (median, range)	44(30-115)	49(30.75-136.75)	65.5(30.5-133.25)	0.675	-0.400	0.997	0.013
ALP (U/L) (median, range)	88(70-125)	89(70.25-132.25)	105(72.25-144.75)	0.376	-0.149	0.526	0.011
ALT (≥39 vs <39 U/ml)	11/60(15.5%)	10/50(16.7%)	4/26(13.3%)	1.000	0.064	0.918	0.026
AST (≥40 vs <40 U/ml)	8/63(11.3%)	8/52(13.3%)	3/27(10.0%)	1.000	0.042	0.649	0.000
Albumin (g/L) (median, range)	43.9(40.5-46.1)	43.5(40.28-45.8)	41.4(39.55-45.5)	0.288	-0.119	0.464	-0.059
Chemotherapy regimens (capecitabine vs GEMOX)	30/41(42.3%)	30/30(50.0%)	19/11(63.3%)	0.053	0.437	0.231	0.129

Bold text hinted that these variables were statistically significant.

P^1^: comparison between CHI group and CH group before PSM.

P^2^: comparison between CHI group and CH group after 1:2 PSM.

CHI group, chemotherapy combined with immunotherapy group; CH group, chemotherapy group; CA19-9, carbohydrate antigen 19-9; HBeAg, hepatitis B e antigen; γ-GT, γ-glutamyl transferase; ALP, alkaline phosphatase; ALT, alanine aminotransferase; AST, aspartate aminotransferase; PSM, propensity score matching; GEMOX, gemcitabine plus oxaliplatin. SMD, standardized mean difference.

### 6 Survival analysis of the CH group and CHI group.

**Figure 5:**
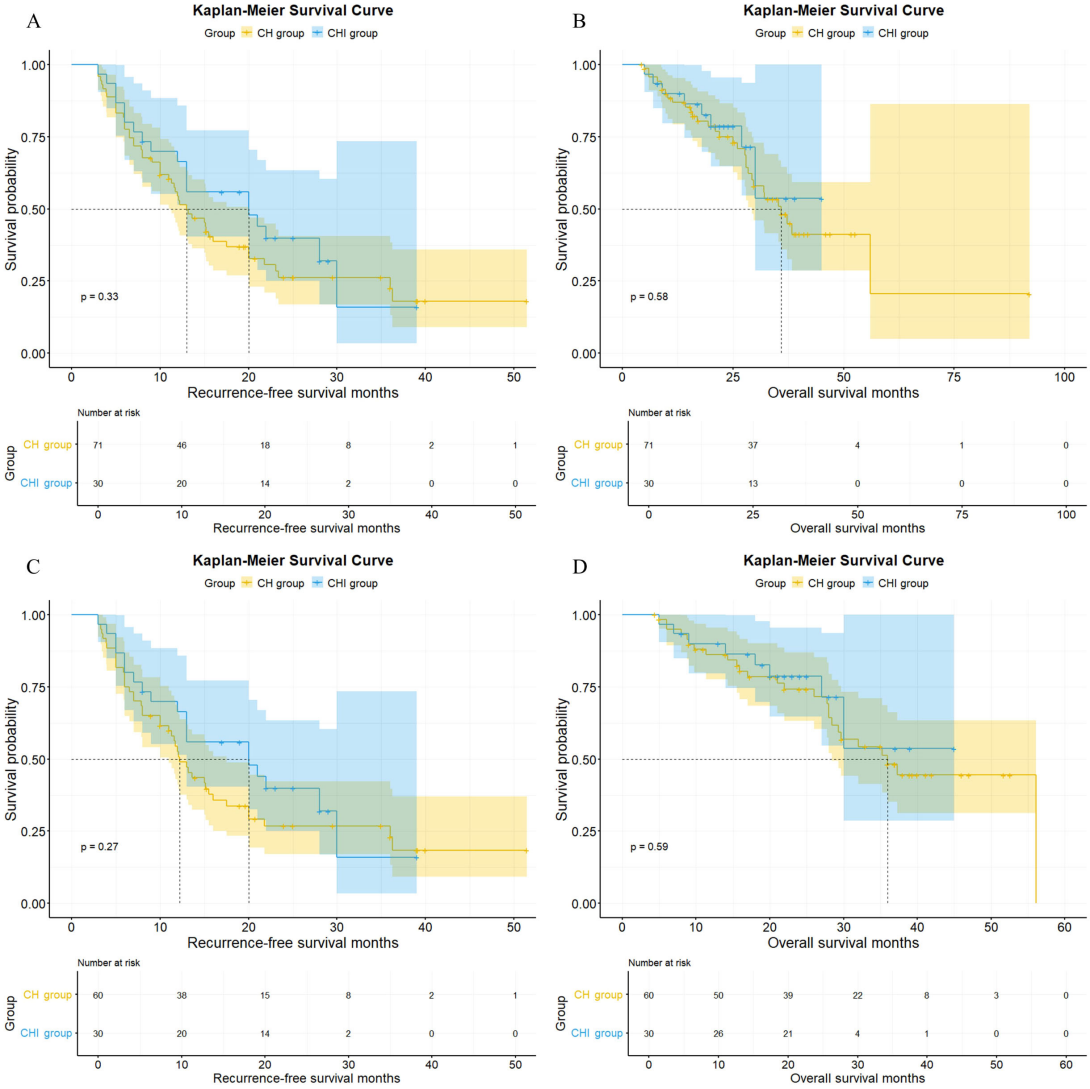
Kaplan-Meier analyses for overall survival (OS) and recurrence-free survival (RFS) between the CH group (n=71) and CHI group (n=30), the CH group (n=60) and CHI group (n=30) after PSM. **(A)** RFS in the CH group versus the CHI group before PSM; **(B)** OS in the CH group versus the CHI group before PSM; **(C)** RFS in the CHI group versus the CH group after 1:2 PSM; **(D)** OS in the CHI group versus the CH group after 1:2 PSM. CH group, chemotherapy group; CHI group, chemotherapy combined with immunotherapy group; PSM, propensity matching scores.

The Kaplan–Meier survival curve is shown in **Figure 5**. Before PSM, RFS was not significantly different between the two groups (p = 0.33), OS was also no significance between the two groups (p = 0.58). Following PSM, no significant difference was observed in both RFS (CH group vs CHI group median RFS: 12.2 vs 20.0 months, p = 0.27) and OS (CHI group median OS has not been reached, CH group median OS: 35.93 months, p = 0.59) between the two groups.

### 7 Primary outcomes in the subgroups.

**Figure 6:**
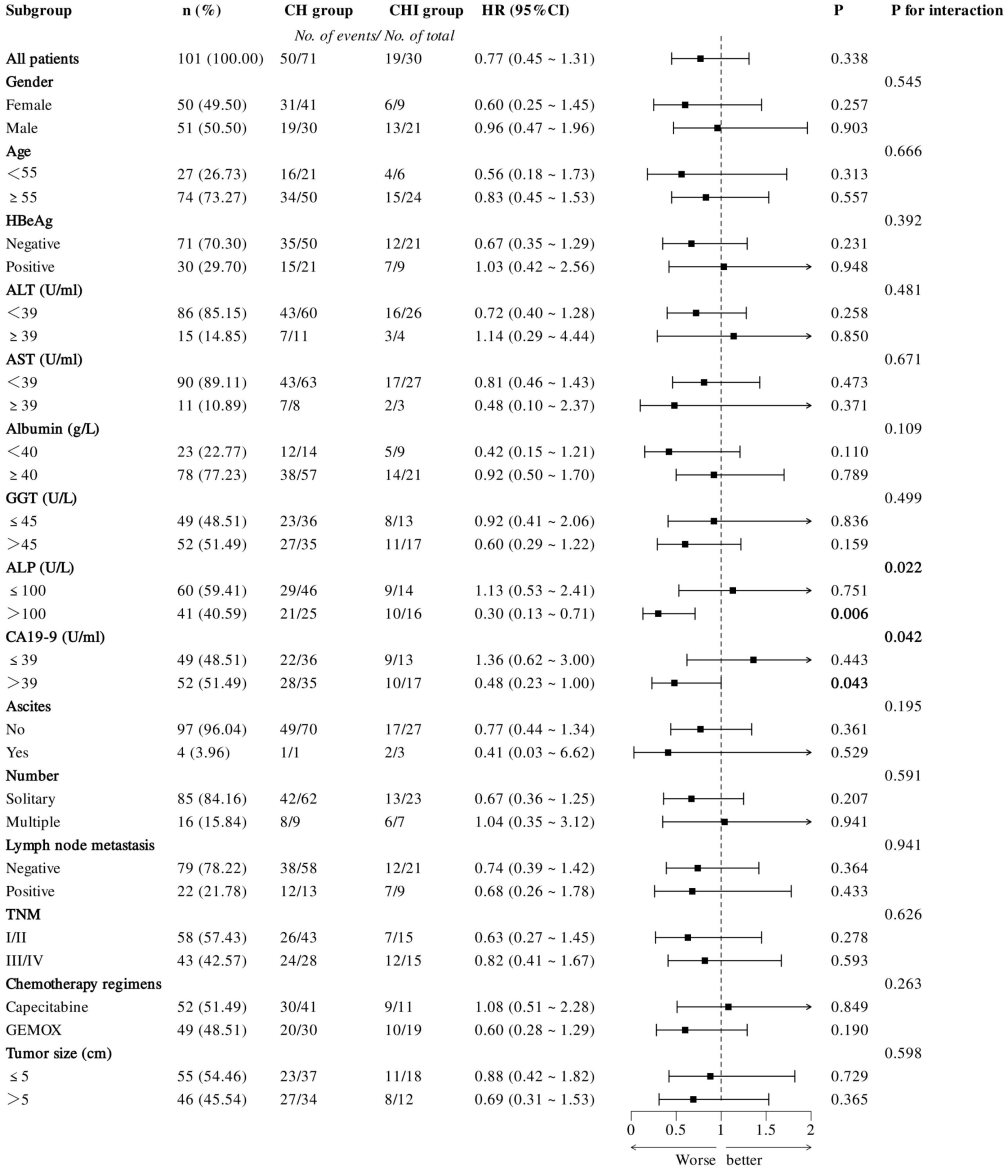
Forest plot of subgroup analysis for recurrence-free survival. HBeAg, hepatitis B e antigen; GGT, γ-glutamyl transferase; ALP, alkaline phosphatase; ALT, alanine aminotransferase; AST, aspartate aminotransferase; CH group, chemotherapy group; CHI group, chemotherapy combined with immunotherapy group.

In exploratory subgroup analysis, chemotherapy combined with immunotherapy was associated with a significant benefit in RFS among patients with CA19-9 >39 U/ml compared to chemotherapy alone (HR, 0.483; 95% CI, 0.233–0.925; p = 0.043), whereas no significant association was observed in patients with CA19-9 ≤39 U/ml (HR, 1.363; 95% CI, 0.619–3.001; p = 0.443; **Figure 6**). The interaction p-value for CA19-9 (>39 vs. ≤39 U/ml) on RFS was 0.042. Similarly, the combination therapy was associated with a significant benefit in patients with ALP >100 U/L (HR, 0.299; 95% CI, 0.126–0.712; p = 0.006), whereas no significant association was observed in patients with ALP ≤100 U/L (HR, 1.131; 95% CI, 0.530–2.410; p = 0.751). The interaction p-value for ALP (>100 vs. ≤100 U/L) on RFS was 0.022.

### 8 Safety of the CH and CHI groups.

**Figure 7:**
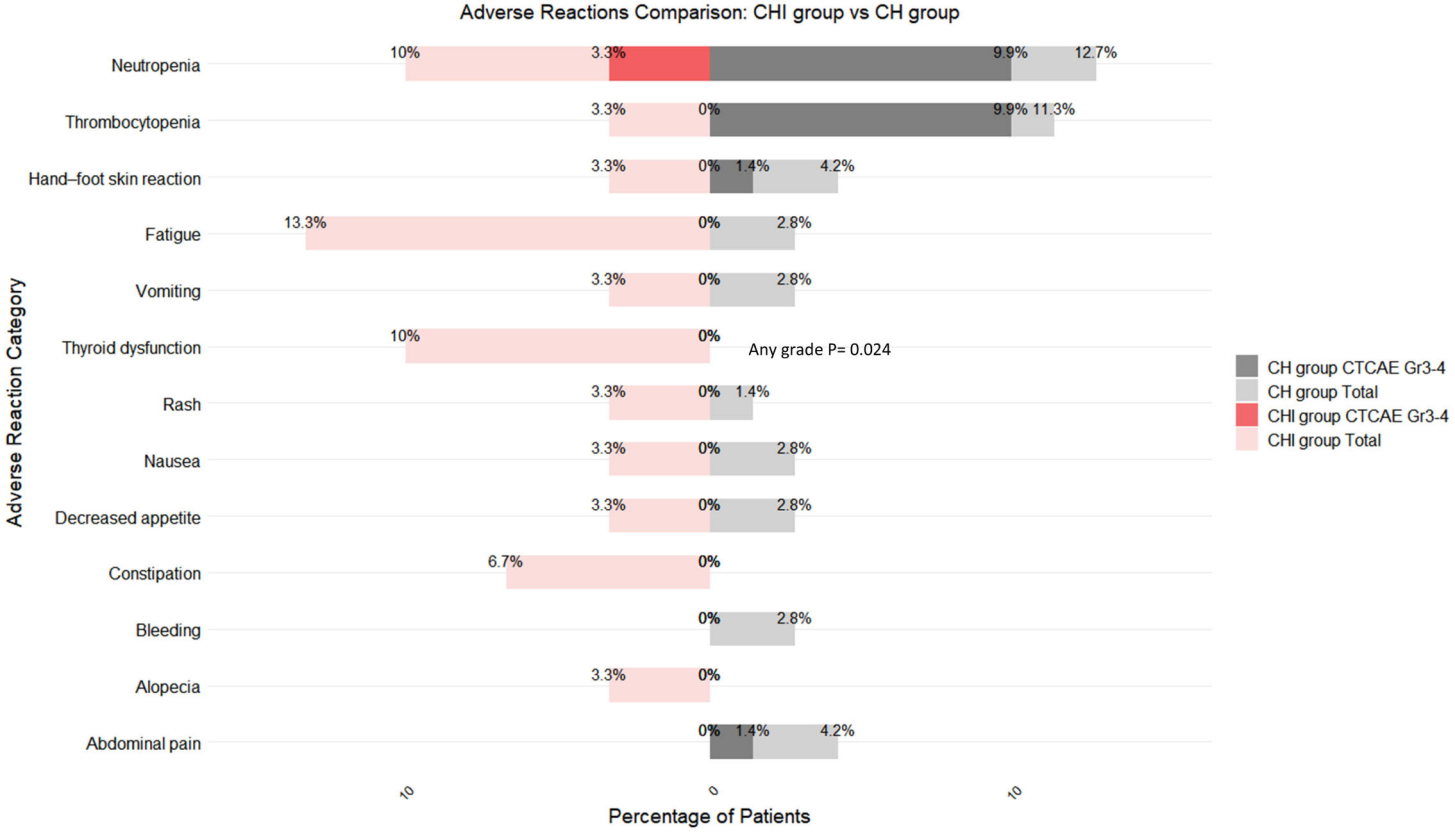
Pyramid plot of adverse events. CH group, chemotherapy group; CHI group, chemotherapy combined with immunotherapy group.

The safety profile of chemotherapy combined with immunotherapy and chemotherapy was assessed through the incidence of adverse event (**Figure 7**; **Supplementary Table 2**).

In the CH group, neutropenia was the most common adverse event (n=9; 12.7%), followed by thrombocytopenia (n=8; 11.3%). Moreover, seven patients developed severe thrombocytopenia and neutropenia with Grade 3-4. One of the patients developed severe hand and foot reactions with grade 3-4. Besides, one patient has abdominal pain with grade 3-4. In the CHI group, fatigue was the most common adverse event (n=4; 13.3%). Neutropenia and thyroid dysfunction were both the second highest incidence of adverse reactions (n=3; 10.0%). One patient has abdominal pain with grade 3-4. There was a statistically significant difference in thyroid dysfunction of any grade between the two groups (p = 0.024). The condition of patients with grade 3-4 adverse reactions improved after drug dosage reduction, drug withdrawal, and symptomatic treatment; and no death owing to such reactions occurred in any group. Details of adjuvant chemotherapy and immunotherapy regimens are shown in **Supplementary Table 3**.

## Discussion

Although surgical resection is currently the only possible cure for ICC, the 5-year OS rate after resection ranging from 25% to 40% (5) for ICC due to its high malignancy.

Some studies have reported that postoperative adjuvant chemotherapy does not benefit patients with ICC (9). The PRODIGE 12-ACCORD 18 (9) study showed that the chemotherapy group did not demonstrate a statistically significant benefit in RFS (HR = 0.88; 95% CI, 0.62-1.25; p = 0.48) and OS (HR = 1.08; 95% CI, 0.70-1.66; p = 0.74). The phase III Bile Cancer Adjuvant Trial (BCAT) (10)showed that adjuvant gemcitabine did not improved RFS or OS. The median RFS for the gemcitabine-treated and observation groups was 36.0 and 39.9months (p = 0.69), respectively, whereas the median OS was 62.3 and 63.8months (p = 0.96). However, the BILCAP study (6) revealed significantly prolonged RFS and OS and was adopted by ASCO (7) recommendations as standard adjuvant therapy. In a network meta-analysis evaluating 1979 patients within six randomized clinical trials (RCT), Salani (11)et al concluded that adjuvant chemotherapy were associated with statistically significant improvements in survival. In another meta-analysis for all BTC, there was also a significant improvement in OS with adjuvant therapy after surgery compared to surgery alone (12). In this study, the CH group exhibited longer RFS (p = 0.045) and OS (p = 0.0084) compared to the non-AT group. The result suggests that adjuvant chemotherapy is significantly beneficial for patients with ICC after radical resection. This aligns with the conclusions of the BILCAP study, while notable differences between the two remain. The BILCAP study includes intrahepatic cholangiocarcinoma, extrahepatic cholangiocarcinoma and gallbladder cancer. An important point to note is that there are significant differences in prognosis among these tumors, which will have an impact on the research results.

We further conducted a comparison between capecitabine and GEMOX. There was no statistical difference between the two groups. In terms of safety, GEMOX group had higher incidence of grade 3-4 adverse reactions(n=7, 17.1%) compared with capecitabine group. In a study by Jang et al. (13), grade 3/4 toxicities included neutropenia (33.9%) and thrombocytopenia (7.6%) were found among patients treated with GEMOX. In the BILCAP trial (6), hand-foot reaction (20%), diarrhea (8%), and fatigue (8%) were the most common adverse events among patients treated with capecitabine monotherapy. The findings of these studies are similar to those of this study. In addition, several clinical trials are actively exploring adjuvant chemotherapy options following resection of ICC. Sun et al. are conducting a clinical trial to evaluate the adjuvant therapy using GEMOX versus capecitabine alone in the ICC patients (14). However, a network meta-analysis evaluating 1308 patients within four RCTs, Akkus (15) et al. concluded that adjuvant 5FU-based monotherapy in resected BTCs provided RFS and OS benefits and gemcitabine-based adjuvant treatment did not provide benefits either in the short (2-year) or long-term outcomes. Despite heterogeneity among trials, this meta-analysis ultimately supports that adjuvant chemotherapy benefits survival outcomes in resected BTC patients.

Current NCCN (16) and ASCO (7) guidelines prioritize capecitabine as the preferred adjuvant therapy. The results of the TOPAZ-1 phase III clinical trial have ushered in a new era of combination immunotherapy and chemotherapy for advanced BTC (17). Several clinical trials have established the efficacy of traditional immune checkpoint inhibitors for the treatment of patients with cancer (18). Moreover, the Phase III KEYNOTE-966 study (19) also demonstrated the promising efficacy of immunotherapy combined with chemotherapy in the treatment of advanced BTC. However, whether this immunochemotherapy combination can provide further benefit in the adjuvant treatment of ICC remains unclear. In this study, we compared the CH group and the CHI group, but detected no significant difference in prognosis between the two groups. However, this finding should be interpreted with caution given the small number of patients receiving immunotherapy (n=30) and the resulting limited statistical power, and these exploratory findings require validation in larger, ideally randomized cohorts. This may be attributed to the following reasons. Firstly, limited overall prognostic benefit: In the KEYNOTE-966 study, gemcitabine and cisplatin (GC) combined with pembrolizumab did not yield a statistically significant progression-free survival (PFS) benefit for patients, and OS at 36 months improved by only 2 percent. Conversely, in the TOPAZ-1 study, GC combined with durvalumab demonstrated a clear PFS benefit, yet the 36-month OS improvement was still only 10%. Collectively, these findings suggest that adding immunotherapy provides limited prognostic improvement for patients with BTC. Secondly, there is a lack of predictive biomarkers. Established biomarkers for predicting response to immune checkpoint inhibitors, such as dMMR/high MSI, are found in only approximately 5%-10% of BTC cases (20). The KEYNOTE-158 phase II trial (NCT02628067) (21) assessed the correlation between PD-L1 expression and the efficacy of pembrolizumab, showing that the overall response rate (ORR) in the BTC cohort was low (5.8%) regardless of PD-L1 expression levels. Furthermore, no statistically significant difference in ORR was observed between PD-L1-positive and PD-L1-negative BTC patients (6.6% vs. 2.9%). Overall, there is currently no ideal biomarker for predicting immunotherapy response in BTC patients. Thirdly, there is a delayed effect of durvalumab in the TOPAZ-1 study. The clinical benefit of durvalumab during the initial 6 months of chemotherapy remains uncertain. The Kaplan-Meier curves for OS and PFS remained largely superimposed for the first 6 months, only separating after chemotherapy. This delayed effect may be associated with enhanced immune activity triggered by antigen release following chemotherapy. In contrast, the tumors in the adjuvant setting were radically resected, potentially reducing antigen availability and consequently weakening the immune response. Interestingly, a phase III trial in gastric cancer (ATTRACTION-5) also failed to demonstrate survival benefit with chemotherapy combined with immunotherapy (22). Dai et al. are conducting a clinical trial to evaluate the efficacy and safety of toripalimab in combination with capecitabine compared to capecitabine monotherapy in the ICC patients (ChiCTR2000036630) (15). Zhou et al. are conducting a Phase II clinical trial to evaluate the efficacy of the PD-1 antibody (SHR-1210) in combination with capecitabine in the ICC patients (NCT04295317) (23). We look forward to the results of these clinical trials.

In exploratory, hypothesis-generating subgroup analysis, chemotherapy combined with immunotherapy was associated with a significant benefit compared with chemotherapy alone in patients with CA19-9 >39 U/ml and ALP >100 U/L, and these results require prospective validation.

As reported in the literature, high serum CA19-9 levels prior to surgery are significantly correlated with poorer survival and more aggressive biological behavior in patients diagnosed with ICC (24–26). Besides, it has been reported that ALP serves as an independent prognostic predictor for OS in ICC patients (27) and is correlated with poorer survival (28). Therefore, there is a need for this combination regimen (chemotherapy combined with immunotherapy) to control tumor growth and prolong patient survival.

Notably, the treatment effect was non-significant in the crude pre-PSM analysis but became significant after matching. This shift likely reflects the removal of baseline confounding; however, it must be interpreted with caution. Propensity score matching reduced the sample size to a small, selected subset, and with a limited number of events, the model may be susceptible to overfitting, potentially overestimating the treatment effect. *Post-hoc* significance in a reduced matched cohort may also represent an inflated or chance finding. Our findings require confirmation in larger, prospective cohorts where sensitivity analyses with alternative methodologies can be properly employed.

This study has several limitations that warrant careful interpretation. First, in addition to its retrospective, single-center design and limited sample size, treatment allocation was non-randomized, which inherently introduces selection bias; patients who received adjuvant therapy may have systematically differed from those who did not in ways not fully captured by our propensity score matching. Second, as with all observational studies of adjuvant therapy, a potential immortal time bias exists because patients had to survive from the date of surgery until the initiation of adjuvant treatment. Third, the treatment regimens were heterogeneous, comprising different chemotherapy backbones (GEMOX vs. capecitabine) and various immune checkpoint inhibitors with differing dosing schedules, which may have confounded the observed associations. Fourth, there was a complete absence of biomarker assessment, such as PD-L1 expression, tumor mutational burden, or microsatellite instability status, precluding any biomarker-stratified analysis beyond the clinical parameters examined. Fifth, the median follow-up of 21 months is relatively short for an adjuvant setting in intrahepatic cholangiocarcinoma, and may not adequately capture late recurrence events, particularly for patients with indolent disease biology. Sixth, although we performed propensity score matching to minimize confounding, the limited number of events raises concern for potential overfitting of the propensity score model. Seventh, the subgroup analyses were exploratory and hypothesis-generating by design and require independent, prospective validation. Additionally, detailed data on dose modifications, treatment delays, and reasons for discontinuation were not systematically recorded, precluding a more granular safety analysis. This should be addressed in prospective studies. A further methodological limitation is that covariates for the propensity score model were selected based on statistical significance in univariable analysis (p < 0.05) rather than being pre-specified on clinical grounds. This data-driven strategy may omit important confounders and introduce residual bias. These limitations underscore the need for adequately powered, randomized, biomarker-enriched prospective trials before any change in clinical practice can be recommended.

## Conclusions

In this retrospective analysis, adjuvant chemotherapy following surgery was associated with improved prognosis in patients with resected ICC. Compared to GEMOX, capecitabine showed no significant difference in prognosis but exhibited a more favorable safety profile. Although the addition of immunotherapy did not confer further benefit in the overall cohort, it was associated with superior efficacy in patients with CA19-9 >39 U/ml or ALP >100 U/L. These findings suggest that adjuvant capecitabine may improve outcomes and support existing guidelines, but this approach requires prospective validation before it can be recommended for routine use.

## Data Availability

The raw data supporting the conclusions of this article will be made available by the authors, without undue reservation.
