# Supplementary material for: Chemotherapy combined with immunotherapy vs. chemotherapy: comparison of safety and efficacy in adjuvant therapy for intrahepatic cholangiocarcinoma
Source: Front Oncol. 2026 Jun 22;16:1764663. doi: 10.3389/fonc.2026.1764663 (PMC13333512; doi:10.3389/fonc.2026.1764663)
Supplement: Supplementary file 1 [file DataSheet1.pdf]

Supplement Table 1: Incidence of adverse events

**Variable**	**GEMOX (n = 41)**		**capecitabine (n = 30)**		**P**	
	Any grade n (%)	Grade 3 or 4 n (%)	Any grade n (%)	Grade 3 or 4 n (%)	Any grade	Grade 3 or 4
Thrombocytopenia	7(17.1)	7(17.1)	1 (3.3)	0 (0.0)	0.153	**0.048**
Neutropenia	8(19.5)	7(17.1)	1 (3.3)	0 (0.0)	0.096	**0.048**
Rash	0(0.0)	0 (0.0)	1 (3.3)	0 (0.0)	0.423	-
Hand–foot skin reaction	0 (0.0)	0 (0.0)	3 (10)	1 (3.3)	0.141	0.423
Abdominal pain	1 (2.4)	0 (0.0)	1 (3.3)	0 (0.0)	1.000	-
Bleeding	0 (0.0)	0 (0.0)	1 (3.3)	0 (0.0)	0.423	-
Nausea	1(2.4)	0 (0.0)	1 (3.3)	0 (0.0)	1.000	-
Decreased appetite	1 (2.4)	0 (0.0)	1 (3.3)	0 (0.0)	1.000	-
Fatigue	0 (0.0)	0 (0.0)	2 (6.7)	0 (0.0)	0.175	-
Vomiting	1 (2.4)	0 (0.0)	1 (3.3)	0 (0.0)	1.000	-

Bold text hinted that these variables were statistically significant.
GEMOX: gemcitabine plus oxaliplatin;

Supplement Table 2: Incidence of adverse events

Variable	CHI group (n = 30)		CH group (n = 71)		P	
	Any grade n (%)	Grade 3 or 4 n (%)	Any grade n (%)	Grade 3 or 4 n (%)	Any grade	Grade 3 or 4
Thrombocytopenia	1(3.3)	0 (0.0)	8 (11.3)	7 (9.9)	0.370	0.176
Neutropenia	3(10)	1(3.3)	9 (12.7)	7 (9.9)	0.965	0.480
Rash	1(3.3)	0 (0.0)	1 (1.4)	0 (0.0)	0.508	-
Hand–foot skin reaction	1(3.3)	0 (0.0)	3 (4.2)	1 (1.4)	1.000	1.000
Abdominal pain	0 (0.0)	0 (0.0)	3 (4.2)	1(1.4)	0.553	1.000
Bleeding	0 (0.0)	0 (0.0)	2 (2.8)	0(0.0)	1.000	-
Nausea	1(3.3)	0 (0.0)	2 (2.8)	0 (0.0)	1.000	-
Decreased appetite	3(3.3)	0 (0.0)	2(2.8)	0 (0.0)	0.308	-
Fatigue	4(13.3)	0 (0.0)	2 (2.8)	0 (0.0)	0.114	-
Vomiting	1(3.3)	0 (0.0)	2(2.8)	0 (0.0)	1.000	-
Thyroid dysfunction	3(10)	0 (0.0)	0 (0.0)	0 (0.0)	**0.024**	-
Alopecia	1(3.3)	0 (0.0)	0 (0.0)	0 (0.0)	0.297	-
Constipation	2(6.7)	0 (0.0)	0 (0.0)	0 (0.0)	0.086	-

Bold text hinted that these variables were statistically significant.

CH group: chemotherapy group;

CHI group: chemotherapy combined with immunotherapy group;

Supplement Table 3: Details of adjuvant chemotherapy and immunotherapy regimens

Variable	Chemotherapy alone (n = 71)	Chemotherapy + immunotherapy (n = 30)
**Chemotherapy backbone, n (%)**		
GEMOX	41 (57.8)	11 (36.7)
Capecitabine	30 (42.2)	19 (63.3)
**Chemotherapy dose**		
Gemcitabine (mg/m²)	1000	1000
Oxaliplatin (mg/m²)	85	85
Capecitabine (mg/m², twice daily)	1250	1250
**Cycles completed, median (range)**	5 (2–8)	4(3–7)
**Treatment duration (months), median (IQR)**	3.5 (1.4–6)	2.8 (2–5)
**ICI type, n (%)**		
Sintilimab (200mg q3w)	—	10 (33.3)
Toripalimab (240mg q3w)	—	11 (36.7)
Tislelizumab (200 mg q3w)	—	5 (16.7)
Camrelizumab (200mg q3w)	—	2 (6.7)
Others	—	2 (6.7)
**Time from surgery to treatment (days), median (IQR)**	42 (22–50)	40 (29–53)

Supplement Figure 1: SMD before and after PSM

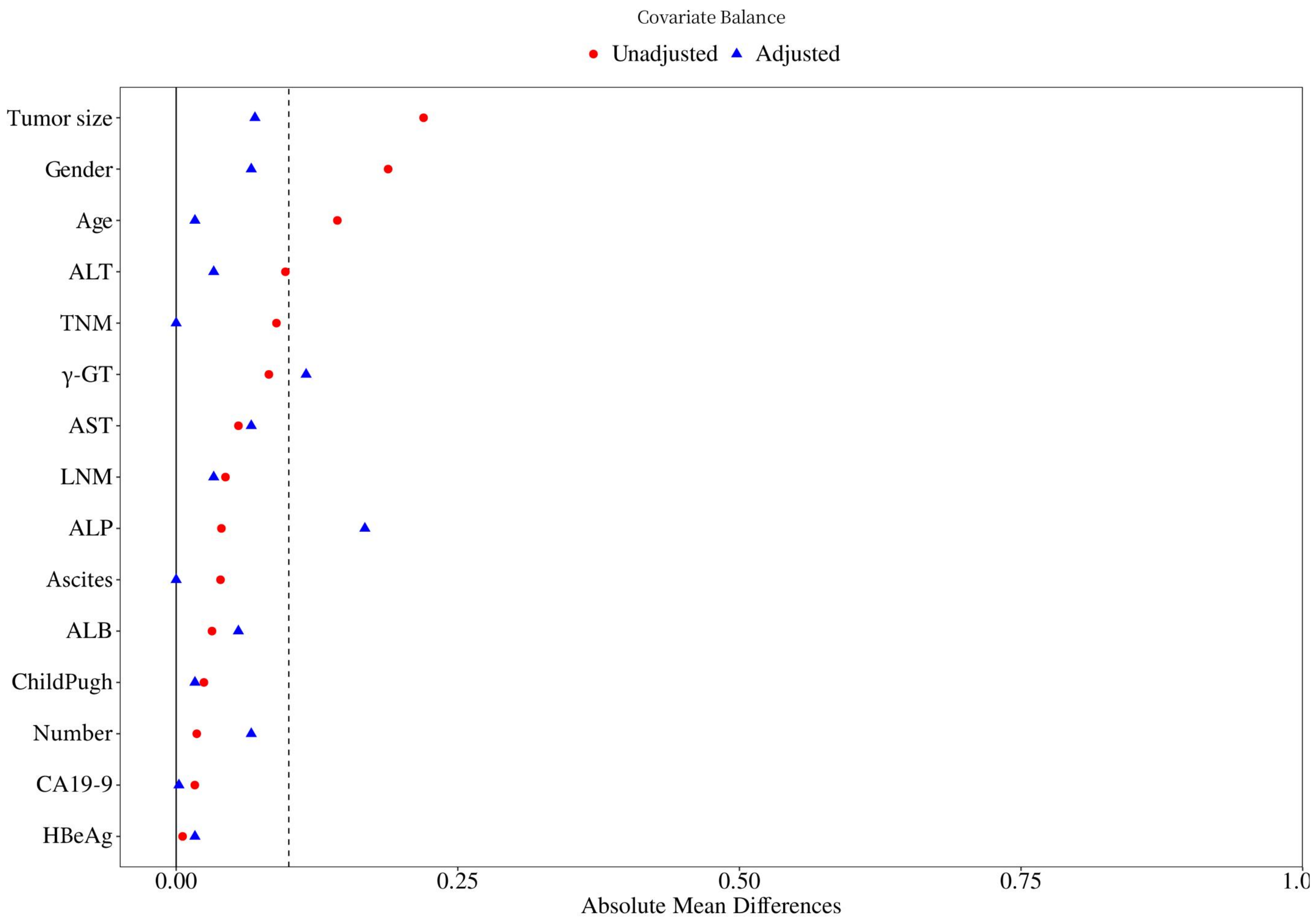

Supplement Figure 2: Density plot of propensity scores before and after propensity score matching.

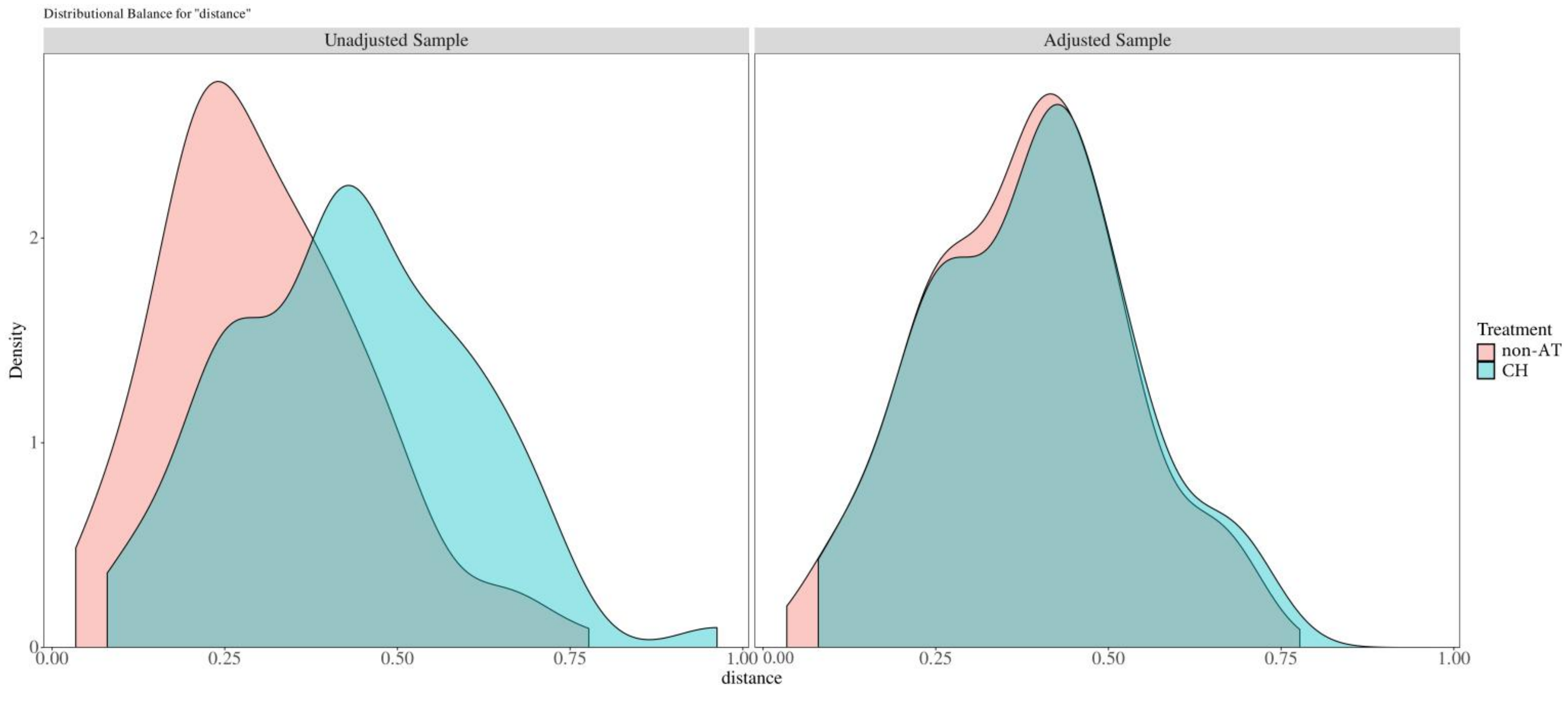

non-AT: patients who received only surgical treatment

CH group: chemotherapy group;

Supplement Figure 3: SMD before and after PSM

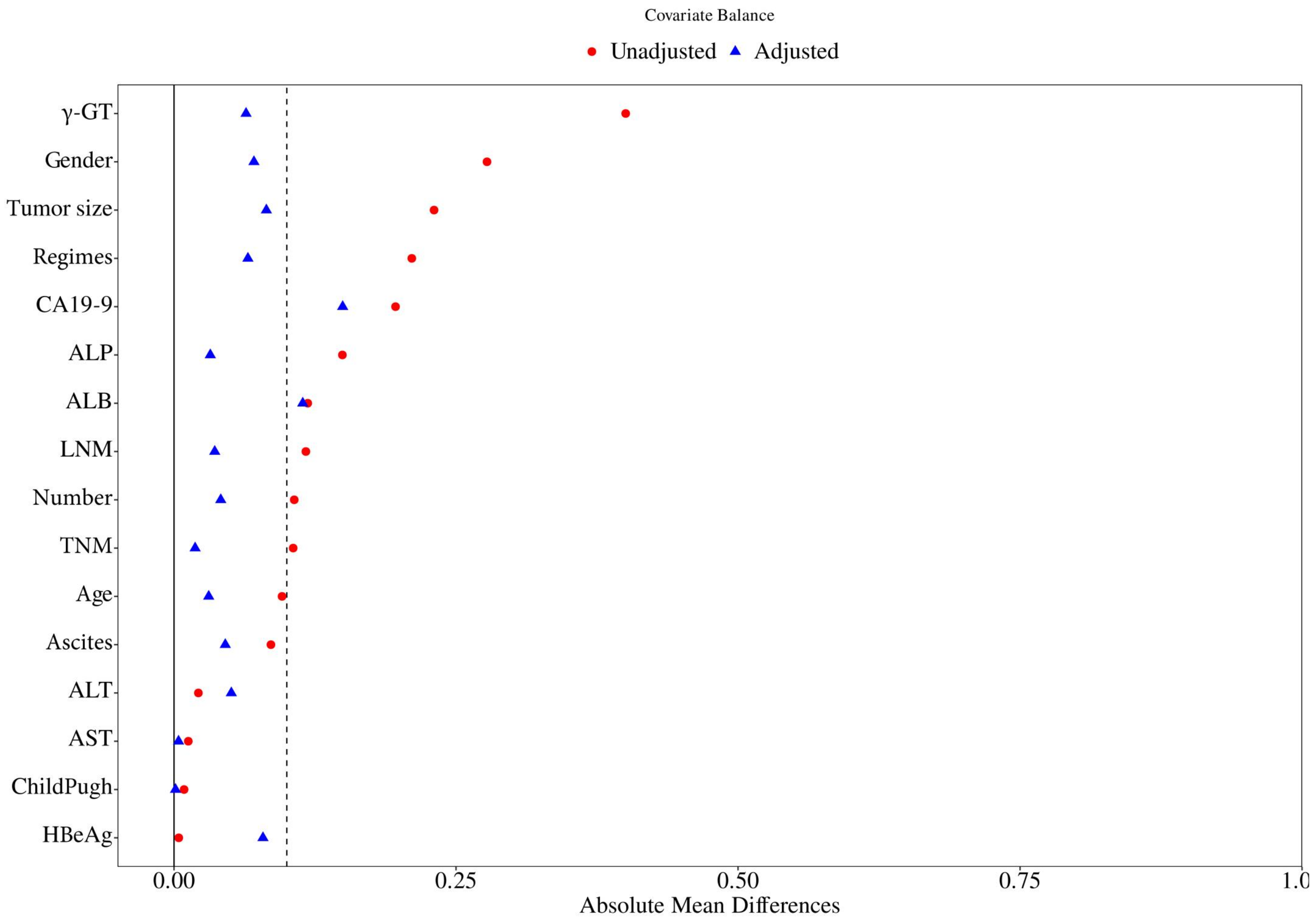

Supplement Figure4: Density plot of propensity scores before and after propensity score matching.

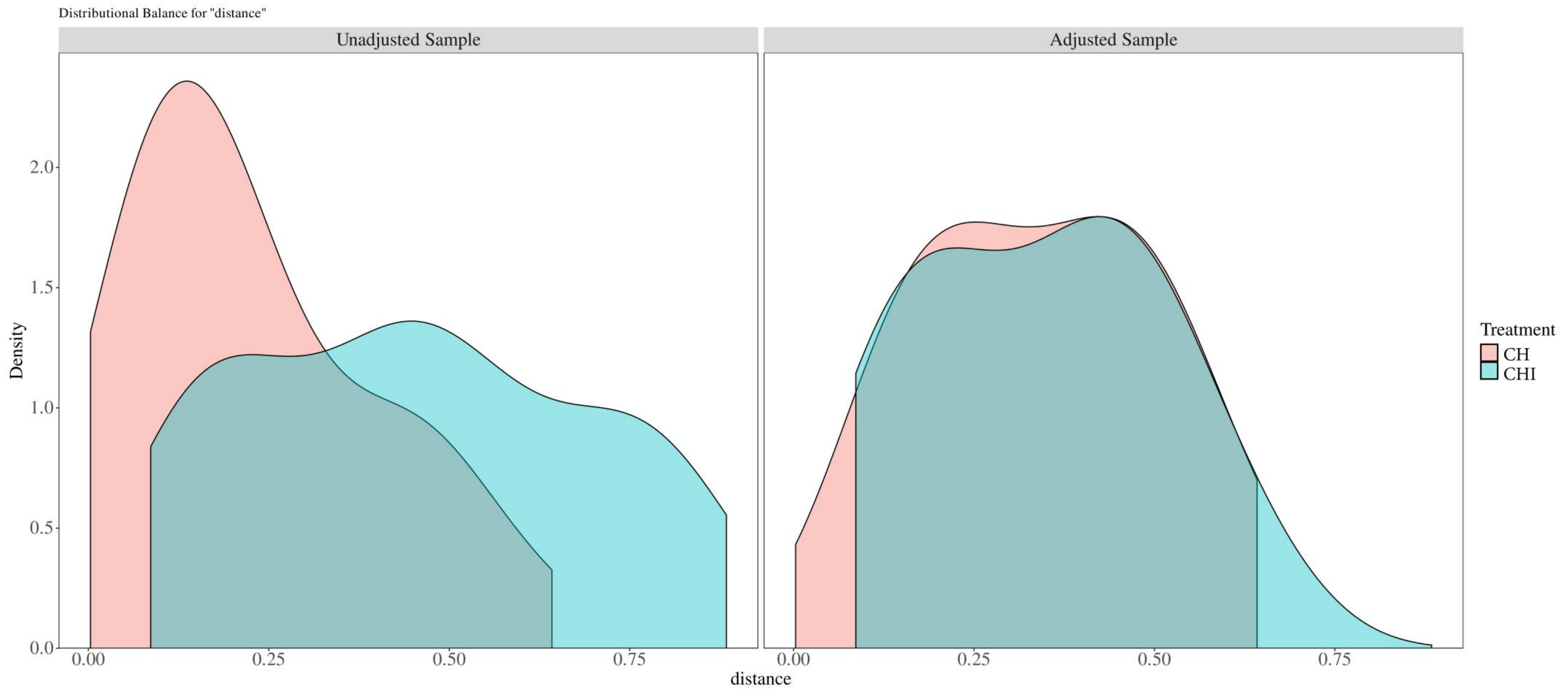

CHI group: chemotherapy combined with immunotherapy group;

CH group: chemotherapy group;
